# Serum and Cerebrospinal Fluid Testing in Optic Neuropathy Patients with Malignant Tumors

**DOI:** 10.1155/2022/7076385

**Published:** 2022-02-17

**Authors:** Chuan-bin Sun, Geng-hao Liu, Qing Xiao, Yi-nv Zhao, Qian Ren

**Affiliations:** ^1^Eye Center, Second Affiliated Hospital of Zhejiang University School of Medicine, Hangzhou 310009, China; ^2^Department of Ophthalmology, Shijiazhuang People's Hospital, Shijiazhuang 050011, China

## Abstract

**Purpose:**

To evaluate the value of serum and cerebrospinal fluid (CSF) testing in optic neuropathy (ON) patients with malignant tumors.

**Methods:**

Fourteen patients clinically diagnosed as ON with malignant tumors but without intracranial or orbital mass in MRI were included in this study. Detailed medical records including medical history, complete ophthalmic examination, colour fundus photography, visual field test, orbital MRI examination, serum and CSF testing data were collected and analyzed. The diagnosis of paraneoplastic optic neuropathy (PON) based on the 2004 recommended criteria of the paraneoplastic syndrome- Euronetwork consortium for paraneoplastic neurological disorders, and current adaption for neuropathies. All patients underwent serum tests for pathogens and autoantibodies including antinuclear antibodies, anticardiolipin antibodies, antineutrophil cytoplasmic antibodies, AQP4-Ab and MOG-Ab, as well as CSF tests for malignant cells under microscope. Serum paraneoplastic antibodies were detected in PON patients. Monkey cerebellar tissue-based assay was used to detect unknown serum anti-neuron antibodies in PON patients with negative paraneoplastic antibody testing results.

**Results:**

Fourteen ON patients were classified as four groups based on their clinical and MRI characteristics, as well as serum and CSF testing results: [1] definite PON, 6 cases (11 eyes); [2] possible PON, 3 case (5 eyes); [3] meningeal carcinomatosis-associated optic neuropathy (MCON), 4 cases (6 eyes); [4] infiltrative optic neuropathy (ION), 2 cases (2 eyes). Malignant cells were found under microscope in CSF samples from MCON and ION patients, contrast to no malignant cells in CSF samples from PON cases. All 14 ON patients with malignant tumors showed negative results in serum tests for pathogens and autoantibodies. Serum paraneoplastic antibodies were tested in PON patients, anti- CV2, anti-Yo, and anti- amphiphysin were detected positive in 2, 1, and 1 case, respectively, in definite PON group, whereas no serum paraneoplastic antibody detected in possible PON group. Two unknown serum antineuronal antibodies (an anti- Purkinje cell antibody and an anti-granular cell antibody) were detected using monkey cerebellar tissue-based assay in 2 of 5 PON patients with negative paraneoplastic antibody test results.

**Conclusions:**

Serum and CSF tests are of great importance in differentiating different subtypes of ON with malignant tumors. Current diagnosis of PON still depends on combination of clinical and MRI manifestations, as well as serum and CSF tests. Tissue-based assay may help to detect new biomarkers for ON etiology and diagnosis.

## 1. Introduction

Optic neuropathy (ON) such as papilloedema secondary to intracranial metastasis is not uncommon in patients with malignant tumors, however, other optic neuropathies such as paraneoplastic optic neuropathy (PON), infiltrative optic neuropathy, and demyelinating optic neuritis, are rare in malignant tumor patients, and their early diagnosis is quite challenging in clinical practice [1–4].

PON, also called paraneoplastic optic neuritis, is a rare but blindness-causing inflammatory disease [1–5]. PON is believed to be caused by the immune-mediated cross-reaction between the malignant tumor and the retina and (or) optic nerve which share same antigens, rather than by an infiltration or metastasis of a malignant tumor. PON should be considered as a possible diagnosis in any cancer patient with optic disc edema and subacute bilateral visual loss, especially when there is no evidence of intracranial or orbital metastasis [1–3].

However, it is really difficult and challenging to distinguish PON from other optic neuropathies including infiltrative optic neuropathy (ION), meningeal carcinomatosis-associated optic neuropathy (MCON), and demyelinating optic neuritis in malignant tumor cases because of many overlapping clinical manifestations [1–6]. We herein evaluated the serum and cerebrospinal fluid (CSF) testing in ON patients with malignant tumors but without intracranial or orbital mass in MRI examination.

## 2. Materials and Methods

### 2.1. Patients

Fourteen patients clinically diagnosed as ON with malignant tumors but without intracranial or orbital mass in MRI from May, 2017 to November, 2021 in Second Affiliated Hospital of Zhejiang University School of Medicine and Shijiazhuang People's Hospital were included in this study. Detailed medical records including medical history, complete ophthalmic examination, colour fundus photography, visual field test, orbital or cranial MRI examination, serum and CSF testing data were collected and analyzed. This study was conducted according to the tenets of the Declaration of Helsinki. Informed consents were obtained from all patients. Institutional review board approvals were obtained from Second Affiliated Hospital of Zhejiang University School of Medicine and Shijiazhuang People's Hospital.

The inclusion criteria were as follows: (1) definite ON diagnosis based on the typical ophthalmic manifestations: (a) acute or subacute visual loss, or blurred vision, (b) swollen optic disc, or occasionally normal optic disc, (c) exclusion of severe cataract, glaucoma, and toxic, compressive, traumatic, or hereditary optic neuropathy; (2) at least one malignant tumor diagnosed before, during or after ON occurrence; (3) serum autoantibody tests including IgM or IgG antibodies to pathogens, antinuclear antibodies, anticardiolipin antibodies, antineutrophil cytoplasmic antibodies, aquaporin 4-IgG antibody (AQP4-Ab), and myelin oligodendrocyte glycoprotein-IgG antibody (MOG-Ab); (4) serum paraneoplastic autoantibody tests in suspected PON patients; (5) at least 6 months follow-up.

The exclusion criteria were: (1) positive serum IgM or IgG antibodies of pathogens including treponema, mycobacterium tuberculosis, herpes viruses, hepatitis viruses, or HIV, which indicating infectious ON; (2) positive serum autoantibodies including antinuclear antibodies, anticardiolipin antibodies, antineutrophil cytoplasmic antibodies, and instant excellent therapeutic response to steroid which supporting a diagnosis of typical inflammation-related ON; (3) concurrent uveitis or retinopathy probably not related to paraneoplastic syndrome; (4) one or more intracranial or orbital mass in MRI.

The diagnosis of PON was based on the 2004 recommended criteria of the paraneoplastic syndrome (PNS)-Euronetwork consortium for paraneoplastic neurological disorders, and the adaption for neuropathies suggested by Antoine et al. Briefly, definite PON was diagnosed based on (1) a direct pathogenic link between the tumor and ON was demonstrated, with or without positive serum paraneoplastic antibodies; (2) well-established PNS but no identified paraneoplastic antibodies, (3) ON unequivocally improved by tumor treatment provided that it has no spontaneous tendency to recovery. Any other ON occurring within 2 years of a cancer was a possible paraneoplastic disorder [1, 2]. ION was defined as optic nerve infiltration by metastatic malignant tumors such as leukemia and lymphoma, or inflammation near optic nerve. MCON was defined as swollen optic disc secondary to meningeal metastasis of malignant tumors such as leukemia, lymphoma, lung and breast cancer. ION can occur alone or accompanied by MCON.

### 2.2. Ophthalmic Examination

All patients underwent best corrected visual acuity (BCVA), complete ophthalmic examination, colour fundus photography, and visual field test was tested using a Snellen chart. Colour fundus photography was taken using Canon CX-1 (Canon Company, Japan), visual field was tested using 30 program for Octopus 900 (HAAG-STREIT Diagnostics, Swissland) perimeter, low vision program was used for patients with BCVA lower than 20/200, but better than hand motion.

### 2.3. Orbital/Cranial MRI Examination

At presentation, all patients underwent orbital or **c**ranial MRI examination using T2-weighted imaging sequence with fat suppression and fluid attenuated inversion recovery sequence, and T1-weighted imaging sequence with fat suppression sequence and gadolinium-enhancement.

### 2.4. Serum and CSF Testing

All patients underwent serum tests including T-spot test, serum IgM or IgG antibodies to pathogens including treponema, mycobacterium tuberculosis, herpes viruses, hepatitis viruses, and HIV, antinuclear antibodies including antinuclear antibody, antidouble-stranded DNA, anti-Sjogren syndrome A or Sjogren syndrome B, anticardiolipin antibodies, antineutrophil cytoplasmic antibodies, serum AQP4-Ab and MOG-Ab tested using cell-based assay. Serum paraneoplastic antibodies were detected using different test panels commercially available predominantly including anti-Hu, anti-Yo, anti-Ri, anti-CV2/CRMP5, anti-Ma2/TA, anti-amphiphysin, anti-Tr, anti- Zic4, and anti-recoverin, in PON patients using indirect immunofluorescence testing and western blotting. Monkey cerebellar tissue -based assay was used to detect unknown serum antineuronal antibodies in PON patients with negative paraneoplastic antibody test results. CSF tests for malignant cells under microscope were performed in all patients.

## 3. Results

### 3.1. Clinical and MRI Characteristics of ON Patients with Malignant Tumors

Table 1 showed the clinical and MRI characteristics, as well as serum and CSF testing results of fourteen ON patients (24 eyes) with malignant tumors but without intracranial or orbital mass in MRI, including nine males and five females. The mean age was 55.6 (range from 37 to 64) years, mean follow-up was 22.5 (range from 6 to 55) months. The concurrent malignant tumors included lung cancer, gastric cancer, prostate cancer, ovarian cancer, nasopharyngeal carcinoma, leukemia, and lymphoma in 7, 2, 1, 1, 1, 1, 1case, respectively. Malignant tumours were treated with at least one of the following: surgery, chemotherapy, radiotherapy, and targeted therapy with biologic agents. Eleven patients with malignant tumors were cured or kept stable at last follow-up, whereas three cases (Case 3, 4, 5) died of relapsed or metastasized malignant tumors.

Fourteen ON patients were classified as four groups based on their clinical and MRI characteristics, as well as serum and CSF testing results: (1) definite PON, 6 cases (11 eyes); (2) possible PON, 3 case (5 eyes); (3) MCON, 4 cases (6 eyes); (4) ION, 2 cases (2 eyes) (Table 1). Nine ON patients (Case 1 to Case 9) were classified as PON based on the direct pathogenic link between the tumor and neuropathy, and ON totally or partially improved after tumor treatment during long-term observation. However, 3 of above 9 PON patients (Case 7 to Case 9) underwent concurrent short-term steroid pulse therapy continued by tapered oral steroid when their ON occurred. More over, these 3 cases were negative for serum paraneoplastic antibody tests. Hence, they were classified as possible PON.

**Figure 1:**
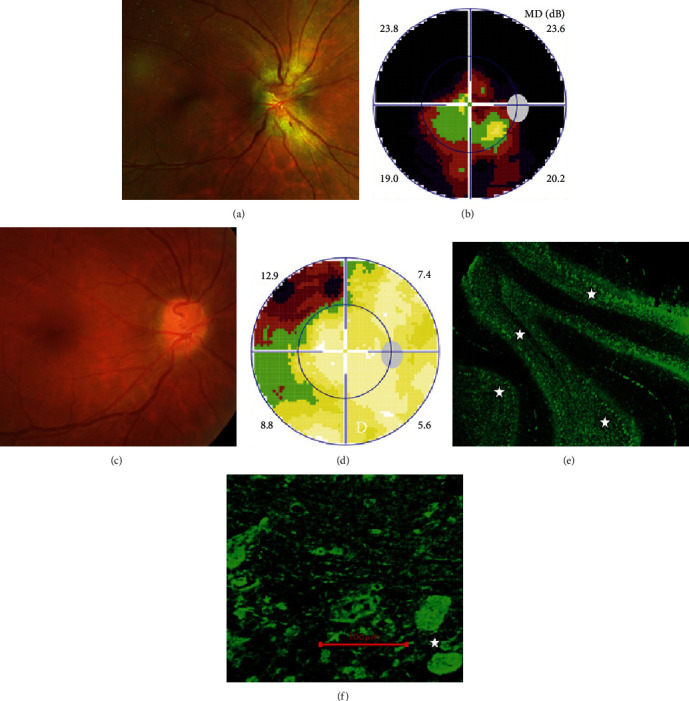
Ophthalmic examination and tissue-based assay of definite paraneoplastic optic neuropathy with lung cancer in Case1. Fundus photograph showed swollen optic disc (a), visual field test showed tunnel vision (b) in the right eye. 2 months after chemotherapy, the edema of optic disc greatly resolved (c) and only small peripheral visual field loss left (d). Unknown serum antineuronal antibody bound to monkey cerebellar Purkinje cells (star, E, F) was detected by tissue-based assay in this patient whose paraneoplastic antibody test result was negative.

**Figure 2:**
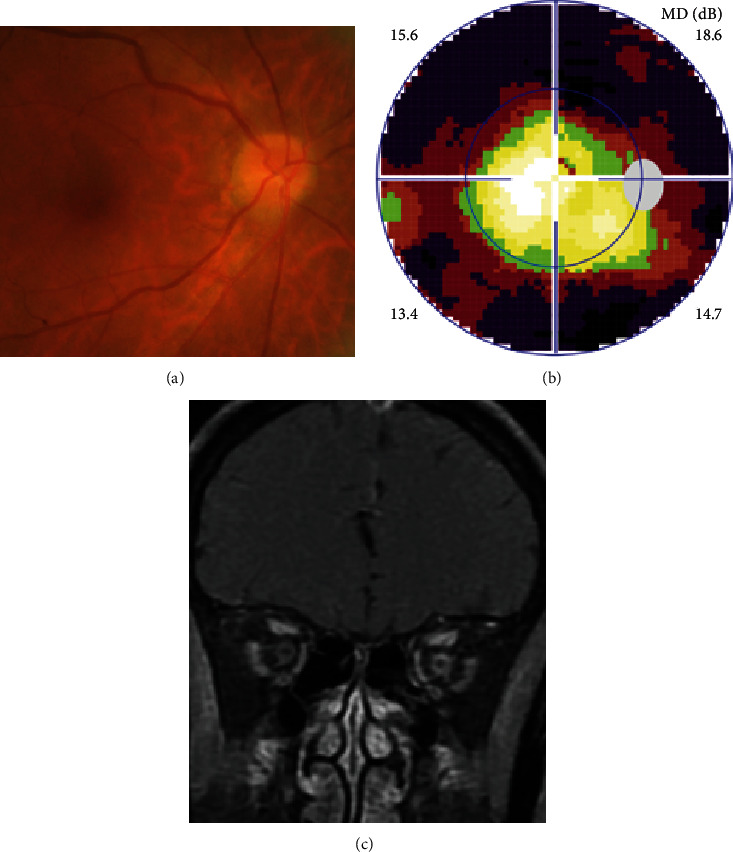
Ophthalmic and MRI examination of definite paraneoplastic optic neuropathy with gastric cancer in Case 4. Fundus photograph showed normal optic disc (a), visual field test showed ring scotoma (b), MRI showed optic nerve enlargement with sheath enhancement in both eyes, more evident in the lfet eye (c).

Definite PON presented as subacute (9/11) or acute (2/11) visual loss, mostly bilaterally invovled (5/6). The median of BCVA was 20/200 (range from hand motion to 20/25) and 20/100 (range from no light perception to 20/20) at presentation and final follow-up, respectively. Seven eyes showed visual improvement whereas 4 eyes visual deterioration at last follow-up. Definite PON predominantly appeared as swollen optic disc (9/11), normal optic disc (2/11) was rare in definite PON patients. Visual field test revealed generalized depression, tunnel vision, ring scotoma, arcuate scotoma, and peripheral defect in 5, 3, 1, 1, and 1 eye, respectively (Figures 1, 2). MRI showed optic nerve enlargement with gadolinium enhancement in 8 eyes, whereas normal MRI in the other 3 eyes (Figure 2). Three of 6 definite PON patients were reported dead at last telephone follow-up.

**Figure 3:**
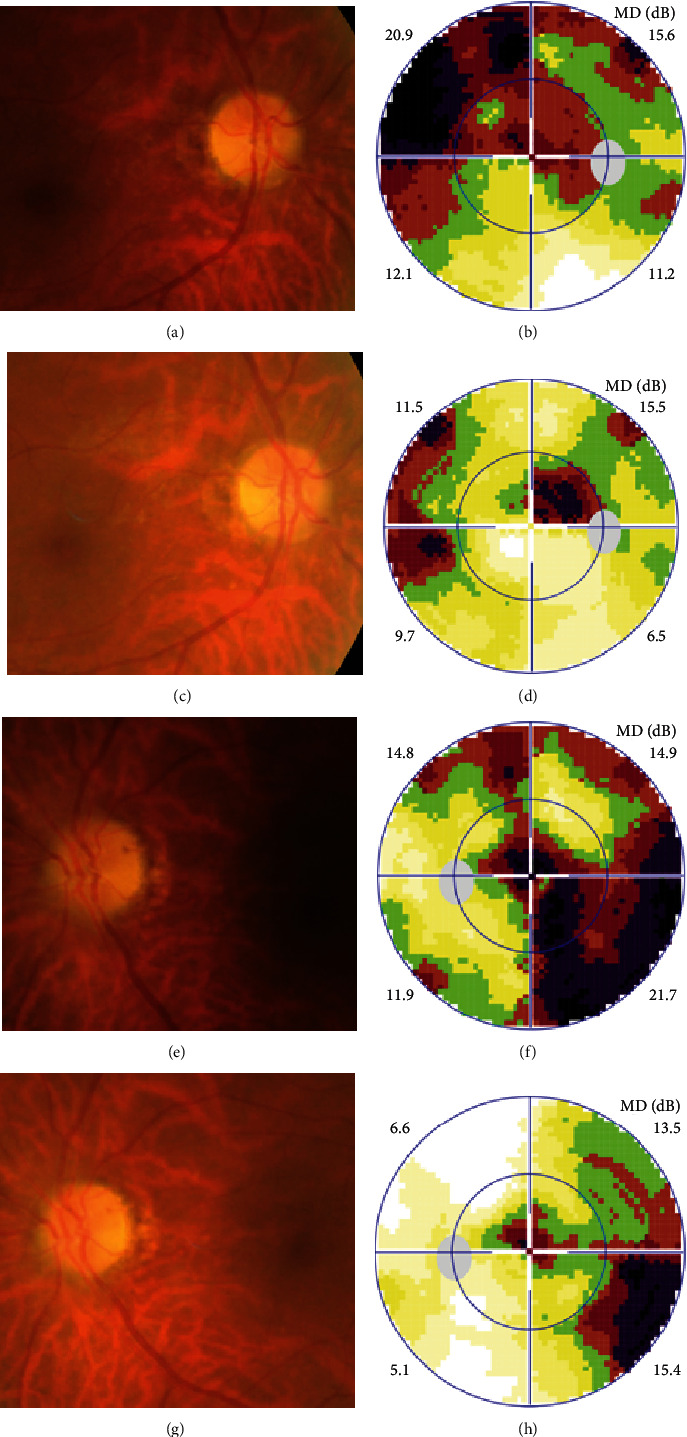
Ophthalmic examination of possible paraneoplastic optic neuropathy with gastric cancer in Case 7. Fundus photographs showed swollen optic disc in both eyes (A, E), visual field test showed altitudinal hemiscotosis (B) and large cental scotoma (F) in the right and left eye. 6 months after surgery and steroid therapy, both eyes appeared as pale temporal disc (C, G), visual field test showed small centrocecal (D) and cental scotoma (H) in the right and left eye, respectively.

Possible PON also presented as subacute (4/5) or acute (1/5) visual loss, also mostly bilaterally invovled (2/3). All possible PON appeared as swollen optic disc, visual field test revealed generalized depression, altitudinal hemiscotosis, and large cental scotoma in 3, 1, and 1 eye, respectively. MRI showed optic nerve enlargement with gadolinium enhancement in one eye, whereas normal MRI in the other 4 eyes (Figure 3). (Table 1).

**Figure 4:**
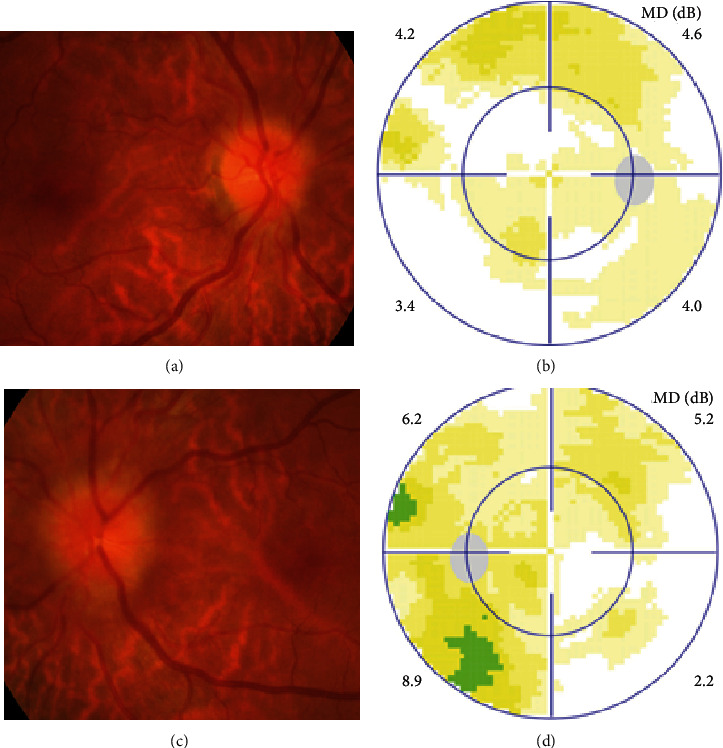
Ophthalmic examination of meningeal carcinomatosis-associated optic neuropathy with gastric cancer in Case10. Fundus photographs showed swollen optic disc (A, C), visual field test was normal (B, D) in both eyes.

**Figure 5:**
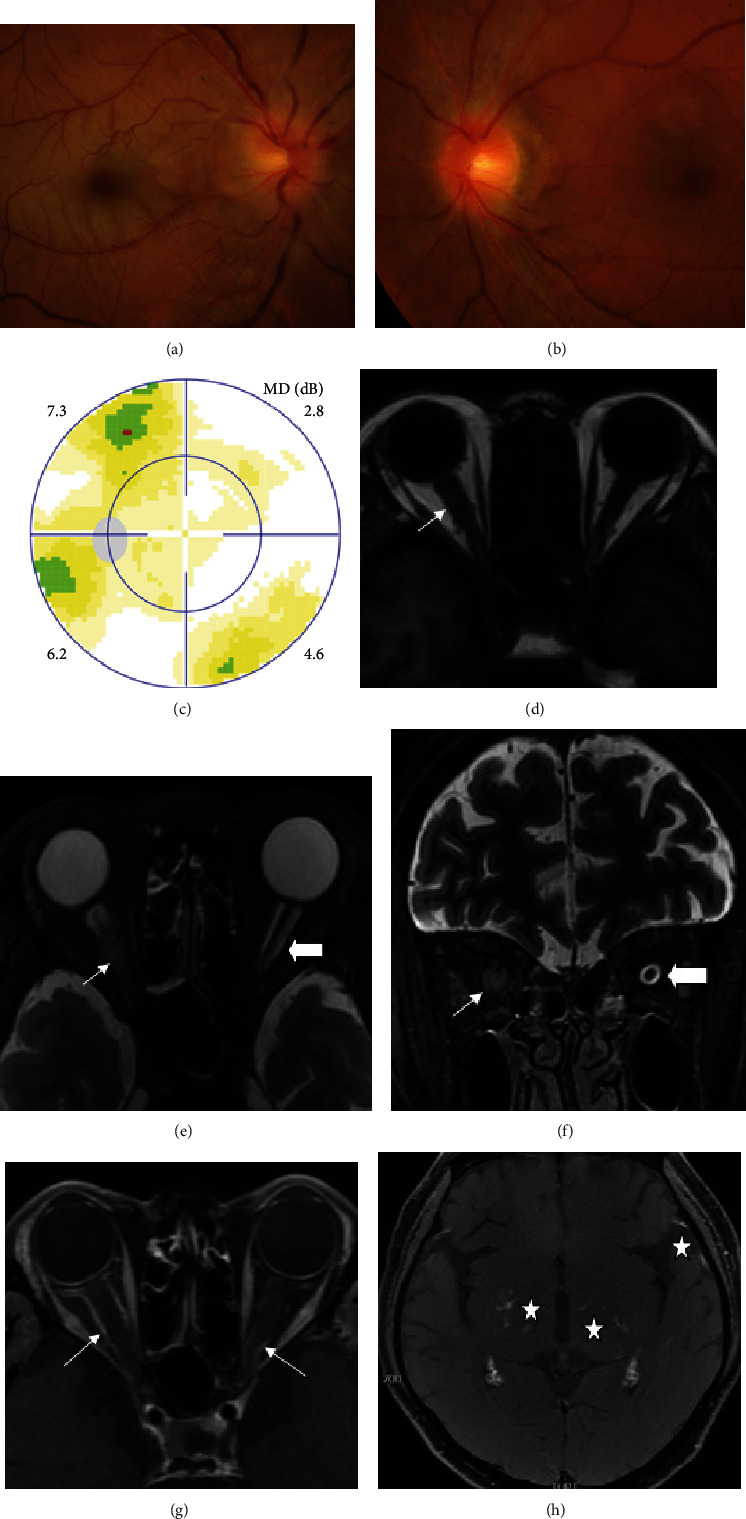
Ophthalmic and MRI examination of infiltrative optic neuropathy (right eye) and meningeal carcinomatosis-associated optic neuropathy (left eye) with leukemia in Case 13. Fundus photographs showed moderate swollen optic disc and posterior retinal edema with macular cherry red in the right eye (A), and mild swollen optic disc in the left eye (B). Visual field test was normal in the left eye (C). MRI showed enlarged optic nerve (short arrow, D, E, F) with sheath enhancement (long arrow, G) in the right eye, enlarged optic nerve sheath space (arrowhead, E, F) with sheath enhancement (long arrow, G) in the left eye. Focal linear enhancement of the meninge, and spotty and linear lesions with enhancement in basal ganglia (star, H) which supporting meningeal carcinomatosis found in T1-weighted MRI with contrast enhancement.

MCON (4 cases, 6 eyes) generally presented as slightly blurred vision, although BCVA and visual field test results are usually normal. Patients with MCON (Case 10 to Case 13) all appeared as swollen optic disc (Figures 4, 5), whereas diplopia due to lateral rectus palsy and enlarged superior ophthalmic vein in B type ultrasound were found in Case 11, and Case 12, respectively. Malignant cell-related cavernous sinus syndrome was found in two cases (Case 11, 12). MRI revealed normal, enlarged optic nerve sheath space and sheath enhancement with or without enlarged cavernous sinus in 2, 3, 1 eye, respectively (Figure 5). However, focal linear enhancement of the meninge which indicating meningeal carcinomatosis was found in T1-weighted MRI with contrast enhancement in all MCON cases (Figure 5).

ION usually presented as subacute progressive visual loss starting from peripheral visual defect, but acute visual loss could also occur when central retinal artery occlusion secondary to ION developed. Patients with ION (Case 13and Case 14) both manifested swollen optic disc, and diffuse posterior retinal edema and cherry-red macular when central retinal artery occlusion occurred. Optic nerve enlargement with sheath enhancement were typical MRI finding of ION (Figure 5).

### 3.2. Serum and CSF Tests of ON Patients with Malignant Tumors

Malignant cells consistent to their primary tumors were found under microscope in CSF samples from MCON and ION patients, although negative results might appear in the first CSF testing. Whereas there was no malignant cells found in CSF samples from PON cases.

All 14 ON patients with malignant tumors included in this study showed negative results in serum tests for pathogens and autoantibodies including antinuclear antibodies, anticardiolipin antibodies, antineutrophil cytoplasmic antibodies, AQP4-Ab and MOG-Ab. Serum paraneoplastic antibodies were tested in PON patients, anti- CV2, anti-Yo, and anti- amphiphysin were detected positive in 2, 1, and 1 case, respectively, in definite PON group, whereas no serum paraneoplastic antibodies detected in possible PON group.

Two unknown serum antineuronal antibodies (an anti- Purkinje cell antibody in Case 1, and an anti-granular cell antibody in Case 8) were detected by monkey cerebellar tissue-based assay in 2 of 5 PON patients with negative paraneoplastic antibody test results (Figure 1).

## 4. Discussion

Ophthalmic involvement including PON and cancer associated retinopathy is common in PNS, even presenting as the initial clinical manifestation of PNS in some cases with malignant tumors [1–4, 7–8]. However, many other optic neuropathies should be excluded before PON was diagnosed in malignant tumor patients with optic nerve involved, which including ischemic anterior ON, papilloedema due to intracranial metastasis, MCON, ION, compressive ON, toxic or malnutritional ON, and demyelinating optic neuritis [1–4, 6–8]. Therefore, early diagnosis of PON is challenging in clinical practice.

Anterior ischemic ON was characterized by mild to moderate visual loss, swollen optic disc spontaneously resolving in 6 to 8 weeks, persistent quadrant or altitudinal hemiscotosis in visual field test, and normal MRI, which could easily differentiated from PON. Toxic or malnutritional ON occurred only after chemotherapy, which could be relieved when chemotherapy stopped.

Papilloedema and compressive ON caused by intracranial or orbital metastasis of malignant tumors could be diagnosed based on one or mass found in MRI. However, early-staged MCON might present as mere swollen disc with no visual defect and normal MRI, at this instance, CSF test for malignant cells was crucial for its diagnosis. Unfortunately, several tests were needed in some cases to find malignant cells in CSF samples.

It was challenging to differentiate PON from ION and demyelinating optic neuritis, because they shared many similar ophthalmic manifestations including acute or subacute visual loss, central or peripheral visual field defect, and optic nerve enlargement with enhancement in MRI [1–6]. CSF test for malignant cells was crucial to exclude ION, and serum autoantibody tests for AQP4-Ab and MOG-Ab were important to exclude AQP4-Ab positive optic neuritis and MOG-Ab related optic neuritis, which was further supported by our results in this study [1–9].

Currently, there still lacks of generally acknowledged criteria for PON diagnosis. The PNS Euronetwork defined six antibodies (anti-Hu, Yo, CV2/CRMP-5, Ri, Ma2 and amphiphysin) as “well characterized onconeural antibodies”, and five antibodies (anti-Tr, ANNA3, PCA2, Zic4, and mGluR1) as “partially characterised onconeural antibodies”, which could confirm or support the diagnosis of PNS [1–3, 10–16]. However, the seropositivity of paraneoplastic antibodies in PON patients is still under evaluation due to lack of enough volume of PON patients [1–4, 8, 10–16]. In our study, serum paraneoplastic antibodies were detected in only 44.4% (4/9) PON patients. The explanation may exist as follows: (1) presently, there is no generally acknowledged technique standards used in serum paraneoplastic antibody testing, seropositivity of paraneoplastic antibodies may differ between agencies supplying commercial testing; (2) maybe there exist other paraneoplastic antibodies responsible for PON still not recognized in laboratory or clinical practice, or tested in paraneoplastic antibody assay kits currently commercially available [17]. Therefore, critical analysis of the causation between the malignant tumor and ON is still of great importance in the diagnosis of PON.

In this study, two unknown serum antineuronal antibodies (an anti-Purkinje cell antibody and an anti-granular cell antibody) were detected by monkey cerebellar tissue-based assay in 2 of 5 PON patients with negative paraneoplastic antibody test results, indicating tissue-based assay may help to detect current or new biomarkers for ON etiology and diagnosis.

There are some limitations in our study. Firstly, the sample size is small, which is common in PNS studies, since PON and PNS are rare in clinical practice. Secondly, the structural characteristics and physiochemical features of two serum anti- neuron antibodies detected by tissue-based assay in PON patients with negative paraneoplastic antibodies test results are still unknown, more workup needs to be done for further understanding the value of these new biomarkers in etiology and diagnosis of optic neuropathies.

## Figures and Tables

**Table 1:** Clinical and MRI characteristics, and serum and CSF test results of optic neuropathy patients with malignant tumors.

No.	Sex	Age	Eye	BCVA		Ophthalmic examination	Visual field	Orbital/cranial MRI	MCs in CSF test	PA in serum test	ON type	Malignant tumor type	Follow-up (months)
				Initial	Final								
1	M	54	R	20/100	20/25	Swollen disc	Tunnel vision	Optic nerve enlargement and enhancement	No	No	dPON	Lung cancer	17
			L	20/40	20/20	Swollen disc	Arcuate scotoma						
2	M	58	R	CF	NLP	Normal disc	Generalized depression	Normal	No	Anti- Amphiphysin	dPON	Lung cancer	10
			L	HM	LP								
3	F	62	R	20/200	20/60	Swollen disc	Generalized depression	Optic nerve enlargement and enhancement	No	Anti-CV2	dPON	Lung cancer	14
			L	CF	20/100								
4	M	62	R	20/25	20/200	Normal disc	Ring scotoma	Optic nerve enlargement and enhancement	No	Anti-CV2	dPON	Lung cancer	6
			L	20/400	NLP	Swollen disc	Tunnel vision						
5	M	61	R	HM	20/125	Swollen disc	Generalized depression	Optic nerve enlargement and enhancement	No	Anti-Yo	dPON	Prostate cancer	8
			L	20/60	20/40	Swollen disc	Tunnel vision						
6	F	48	R	20/60	20/32	Swollen disc	Peripheral defect	Normal	No	No	dPON	Ovarian cancer	49
7	M	61	R	20/200	20/40	Swollen disc	Altitudinal hemiscotosis	Normal	No	No	pPON	Gastric cancer	55
			L	20/100	20/32	Swollen disc	Large cental scotoma						
8	M	54	R	20/100	20/32	Swollen disc	Generalized depression	Normal	No	No	pPON	Lung cancer	20
			L	CF	20/200								
9	F	64	L	CF	20/125	Swollen disc	Generalized depression	Optic nerve enlargement and enhancement	No	No	pPON	Lung cancer	16
10	M	59	R	20/20	20/20	Swollen disc	Normal	Normal optic nerve, focal meningeal enhancement	Yes	N.A.	MCON	Gastric cancer	15
			L	20/20	20/20								
11	F	52	L	20/20	20/20	Swollen disc and lateral rectus palsy	Normal	Enlarged optic nerve sheath space with sheath enhancement, enlarged cavernous sinus with meningeal enhancement	Yes	N.A.	MCON CSS	Lung cancer	6
12	F	57	R	20/50	20/40	Swollen disc Enlarged SOV	Normal	Enlarged optic nerve sheath space with sheath enhancement, enlarged cavernous sinus with meningeal enhancement	Yes	N.A.	MCON CSS	Nasopharyngeal carcinoma	48
			L	20/40	20/32								
13	M	37	L	20/20	20/20	Swollen disc	Normal	Enlarged optic nerve sheath space with sheath enhancement, focal meningeal enhancement	Yes	N.A.	MCON	Leukemia	36
			R	20/40	NLP	Swollen disc CRAO	N.A.	Optic nerve enlargement with sheath enhancement, focal meningeal enhancement	Yes	N.A.	ION		
14	M	49	R	NLP	NLP	Swollen disc CRAO	N.A.	Optic nerve enlargement with sheath enhancement	Yes	N.A.	ION	Lymphoma	15

BCVA: best corrected visual acuity; R: right; L: left; MCs: malignant cells; CSF: cerebrospinal fluid; PA: paraneoplastic antibody; ON: optic neuropathy; CF: counting fingers; HM: hand motion; LP: light perception; NLP: no light perception; PON: paraneoplastic optic neuropathy; dPON: definite PON; pPON: possible PON; MCON: meningeal carcinomatosis-associated optic neuropathy; CSS: cavernous sinus syndrome; SOV: superior ophthalmic vein; ION: infiltrative optic neuropathy; CRAO: central retinal artery occlusion; N.A.: not applicable.

## Data Availability

The data used to support the findings of this study are included within the article.
